# *Gomphus floccosus* (Schw.) Sing. extract attenuates alcoholic liver disease by suppressing macrophage glycolysis and M1 polarization

**DOI:** 10.3389/fimmu.2026.1772592

**Published:** 2026-02-17

**Authors:** Tianyin Ruan, Xutao Li, Mingyue Li, Siyuan Wang, Li Shen, Chenghai Liu, Yuan Peng, Yanyan Tao

**Affiliations:** 1 Institute of Liver Diseases, Shuguang Hospital Affiliated to Shanghai University of Traditional Chinese Medicine, Shanghai, China; 2 Key Laboratory of Liver and Kidney Diseases (Ministry of Education), Shuguang Hospital Affiliated to Shanghai University of Traditional Chinese Medicine, Shanghai, China; 3 Shanghai Key Laboratory of Traditional Chinese Clinical Medicine, Shanghai, China

**Keywords:** alcoholic liver disease (ALD), *Gomphus floccosus* (Schw.) Sing., immunometabolism, M1 polarization, macrophage glycolysis, metabolic reprogramming

## Abstract

**Background:**

Alcoholic liver disease (ALD) remains a leading cause of global mortality, yet the development of safe and effective multi-target therapies continues to be a significant challenge. Macrophage-mediated inflammation plays a pivotal role in the pathogenesis of ALD, with macrophage glycolysis reprogramming emerging as a critical immunometabolic checkpoint that drives disease progression. *Gomphus floccosus* (Schw.) Sing (*Gf*), a mushroom traditionally employed in southwestern China for the treatment of hepatobiliary disorders, holds therapeutic potential. However, its clinical application is limited by gastrointestinal side effects, and its active components and underlying mechanisms in ALD remain largely unexplored. This study aims to determine whether a standardized extract of *Gf* alleviates ALD by specifically modulating the macrophage glycolysis-M1 polarization axis.

**Methods:**

Ultra-high-performance liquid chromatography-mass spectrometry (UHPLC-MS) was utilized to characterize the chemical profile of the *Gf* extract. An *in vivo* ALD mouse model was established using the Lieber-DeCarli ethanol diet with two distinct administration routes. *In vitro* studies were conducted using lipopolysaccharide (LPS)/ethanol-stimulated macrophages (RAW264.7 and THP-1 cell lines). Comprehensive analyses, including transcriptomic sequencing, pathway enrichment studies, and validation through immunohistochemistry, immunofluorescence, qRT-PCR, Western blotting, and metabolic flux analysis, were performed to elucidate the underlying mechanisms.

**Results:**

Three major constituents were identified in the *Gf* extract. Treatment with *Gf* extract significantly mitigated ALD pathology, as evidenced by reductions in steatosis, oxidative stress, and inflammation. Transcriptomic analysis identified 231 differentially expressed genes, with significant enrichment in the glycolysis pathway. Mechanistically, the *Gf* extract suppressed key glycolytic enzymes, including GLUT1, GCK, HK2, PKM2, and LDHA, as well as lactate production in macrophages. This inhibition effectively reduced pro-inflammatory cytokine secretion, chemotaxis, and M1 polarization.

**Conclusion:**

The hepatoprotective effects of *Gf* extract against ALD are mediated through the suppression of macrophage glycolytic reprogramming and M1 polarization, providing an immunological basis for its traditional use in hepatobiliary disorders.

## Highlights

- *Gf* extract represents a promising therapeutic candidate for alcoholic liver disease (ALD).
- An optimized preparation of *Gf* eliminates toxicity while retaining its efficacy against ALD.
- *Gf* extract alleviates ALD by targeting five key glycolytic enzymes in macrophages.

## 1 Introduction

Alcoholic liver disease (ALD) is a significant global health challenge, characterized by a progressive spectrum of liver damage ranging from steatosis to cirrhosis and hepatocellular carcinoma (HCC) (1). According to reports from the World Health Organization (WHO), alcohol-related deaths reached 2.6 million in 2019, and ALD cases surged to 3.02 million in 2021, marking a 38.68% increase since 2000 (2, 3). While alcohol abstinence remains the cornerstone of ALD management and corticosteroids are employed in severe cases, their therapeutic efficacy is often suboptimal. This underscores the urgent need for innovative and effective treatment strategies (1).

Medicinal mushrooms have shown significant potential for multi-targeted interventions in ALD (4, 5). For instance, *Ganoderma lucidum* (Lingzhi) exhibits comprehensive hepatoprotective effects against various forms of liver injury, including ALD (5). Its active compounds, such as polysaccharides, triterpenoids, and ganoderic acid A (GAA), mitigate ALD pathogenesis by reducing hepatic oxidative stress, enhancing ethanol metabolism, and modulating gut microbiota composition (6–8). Similarly, *Poria cocos* (Fuling) is a key component in several validated anti-ALD formulations, highlighting its critical role in traditional hepatoprotective approaches (9).

The pathogenesis of ALD involves both direct hepatocyte damage caused by alcohol metabolism and secondary dysfunction of the intestinal barrier. Alcohol is metabolized into acetaldehyde, which, together with the induction of cytochrome P450 2E1 (CYP2E1), generates significant oxidative stress. This oxidative stress directly damages hepatocytes, initiating hepatic steatosis (10–12). Simultaneously, alcohol compromises intestinal integrity, resulting in increased translocation of gut-derived endotoxins, primarily lipopolysaccharide (LPS), into the portal circulation (11).

Hepatic macrophages, particularly Kupffer cells, become sensitized to these circulating LPS molecules through pattern recognition receptors, such as TLR4. This activation is closely linked to immunometabolic reprogramming, wherein LPS signaling induces a metabolic shift in macrophages from oxidative phosphorylation to aerobic glycolysis, also known as the Warburg effect. This metabolic reprogramming provides a rapid supply of ATP and biosynthetic precursors, driving polarization toward the pro-inflammatory M1 phenotype. M1 macrophages are characterized by robust cytokine production, including IL-1β, which exacerbates liver injury (13).

While alcohol exposure enhances M1 polarization, it concurrently impairs the alternative, pro-repair M2 macrophage polarization. Consequently, alcohol disrupts the balance between M1 and M2 macrophages by not only providing a persistent polarizing stimulus (LPS) but also promoting the metabolic programming necessary to sustain the inflammatory M1 state. This disruption leads to an accumulation of M1 macrophages and persistent inflammation, further aggravating liver damage (14–16).

Macrophage polarization is intricately linked to metabolic reprogramming (17). LPS-induced M1 macrophages exhibit a pronounced reliance on glycolysis, which provides the energy and biosynthetic precursors necessary to sustain their pro-inflammatory phenotype (18). In contrast, M2 macrophages primarily depend on oxidative phosphorylation. Recent studies have highlighted that M1 polarization is critically dependent on glycolytic reprogramming, with evidence showing that inhibition of glycolysis effectively suppresses pro-inflammatory activation (19). Key enzymes involved in this process, including GLUT1, GCK, HK2, PKM2, and LDHA, operate as a coordinated network and represent promising targets for ALD intervention. However, natural products capable of specifically modulating this glycolytic network remain largely unexplored.

Gomphus floccosus (Schw.) Sing., a fungal species traditionally used in folk medicine for the treatment of hepatobiliary disorders, contains a diverse array of bioactive compounds, including alkaloids, terpenoids, saponins, fatty acids, polysaccharides, and peptides. These constituents have demonstrated antifungal (20) and antioxidant (21) activities. However, the presence of toxic compounds such as Norcaperatic acid and Agaricic acids has been associated with gastrointestinal (22) and central nervous system toxicity (23), necessitating the development of refined extracts that eliminate these toxic components while retaining the hepatoprotective fractions.

This study was therefore designed to investigate the mechanism of action of a purified *Gf* extract in ALD models, with a particular focus on its ability to suppress key glycolytic enzymes and attenuate M1 macrophage polarization. Our findings aim to provide experimental evidence supporting the traditional use of *Gf* and contribute to the development of glycolysis-targeted therapeutic strategies for ALD.

## 2 Materials and methods

### 2.1 Preparation and chemical profiling of *Gf* extract

The fruiting bodies of Gomphus floccosus (*Gf*), collected from Yunnan Province, China, were authenticated, and a voucher specimen (No. 251822) was deposited at the Fungarium (HMAS), Institute of Microbiology, Chinese Academy of Sciences. The fungal material used in this study, also sourced from Yunnan, was taxonomically identified by the Testing Department of the Shanghai Traditional Chinese Medicine Standardization Research Center (Testing No. JC21001).

The *Gf* extract (Batch No. 20220921) was prepared through sequential extraction of the dried fungal powder (40 mesh) using 95% and 70% ethanol. The combined extracts were concentrated under reduced pressure to obtain a primary active fraction, which was dissolved in water, filtered, and subjected to sequential liquid-liquid extraction with petroleum ether and ethyl acetate. The final aqueous phase was further purified using D101 macroporous resin, with water as the eluent, and concentrated to yield the final extract. Qualitative and quantitative analyses of the representative chemical constituents in the *Gf* extract were conducted using ultra-high-performance liquid chromatography coupled with Q-Exactive Orbitrap high-resolution mass spectrometry (UHPLC-Q-Exactive Orbitrap HRMS).

### 2.2 Animals

Eight-week-old male C57BL/6J mice (21 ± 1g) were obtained from Shanghai Jihui Experimental Animal Breeding Co., Ltd. (SCXK 2022-0009) and housed in the Specific Pathogen-Free (SPF) facility at Shanghai University of Traditional Chinese Medicine (SYXK 2020-0009). All animals were maintained under controlled environmental conditions. The experimental protocols were approved by the Institutional Animal Ethics Committee (Approval Nos. PZSHUTCM2312250011, PZSHUTCM250210001).

#### 2.2.1 Concurrent administration: 3-week modeling with concurrent treatment

The conventional adult dosage of *Gf* is roughly 10 grams of raw material daily. The yield of the water-soluble *Gf* extract was 14.64%, and the mouse equivalent dose (MED) was determined using a conventional body surface area conversion factor. The formula utilized was: MED (g extract/kg) = (10 g crude drug/70 kg adult) × (conversion factor 10) × (extraction yield 14.64%) ≈ 0.209 g/kg. Considering the potential loss of bioactive components during extraction, the 3-fold (0.628 g/kg) and 6-fold (1.255 g/kg) MEDs, which showed no toxicity in normal mice, were selected as the treatment doses for this study.

Subsequently, 54 mice were randomly allocated into five groups: Normal control (N) (n=8), Alcohol model (M) (n=12), *Gf* low dosage (L, 0.628 g/kg) (n=12), *Gf* high dose (H, 1.255 g/kg) (n=11), and positive control (PPC, 0.195 g/kg Polyene Phosphatidylcholine Capsules) (n=11). The experimental groups of mice were acclimatized to the Lieber-DeCarli ethanol diet (Beijing Xiao Shu You Tai Biotechnology Co., Ltd., China) over one week, with ethanol concentration gradually elevated from 10 mL/L (days 1-3) to 30 mL/L (days 4-6), and subsequently sustained at 57.3 mL/L from day 7 onward. The ethanol diet was subsequently extended for an additional two weeks. During the whole research duration, the control group was sustained on the isocaloric Lieber-DeCarli control diet. All pharmacological therapies were provided during the last week (the third week) in conjunction with ongoing ethanol administration (24).

#### 2.2.2 Post-modeling administration: 3-week modeling followed by 1-week treatment

A second cohort of 40 mice was organized in the same manner as the first cohort (n = 8 per group). Alcohol administration was performed as outlined in section 2.2.1 for a duration of three weeks. Subsequently, all mice were transitioned to the isocaloric control diet, while the treatment groups were administered their designated medications for one week (1).

### 2.3 Biochemical analysis of serum and liver tissue

Biochemical studies of serum and liver homogenates were conducted in accordance with commercial kit methods. Serum alanine aminotransferase (ALT) and aspartate aminotransferase (AST) levels were measured utilizing a microplate assay with 10 μL of serum. For hepatic lipid evaluation, liver tissue was homogenized in anhydrous ethanol, and the amounts of TC(A111-1-1) and TG(A110-1-1) in the resultant extracts were quantified. Moreover, tissue (100 mg) was homogenized in physiological saline to evaluate oxidative stress indicators (GSH, T-SOD, MDA)(A006-2, A001-3; S0131S, Beyotime, China) and glycolytic metabolites (lactate, pyruvate)(A019-2-1, A081-1-1) in the supernatant. All commercial kits were sourced from the Nanjing Jiancheng Bioengineering Institute (China), with the exception of the MDA kit.

### 2.4 Histopathological evaluation

Hepatic histopathology was evaluated on tissue sections stained with H&E and Oil Red O according to recognized techniques. Lipid accumulation was assessed by determining the area percentage of Oil Red O-positive lipid droplets from recorded pictures utilizing ImageJ software (v1.8.0) (25).

### 2.5 Immunohistochemistry

Tissue was deparaffinized and rehydrated. Sections underwent heat-induced epitope retrieval in citrate buffer (7–8 min at boiling, then 15 min at sub-boiling). Sections were cooled to room temperature, washed with TBST and incubated with 3% H_2_O_2_ in methanol to quench endogenous peroxidase. They were then blocked with 5% BSA for 30 min and incubated with primary antibody (30 μL/section) overnight at 4 °C. The next day, sections were washed and incubated with HRP-conjugated secondary antibody (1 h at RT), followed by 20-min SABC reagent incubation. DAB was used to develop color. Sections were counterstained with hematoxylin, dehydrated, cleared and mounted. The immunohistochemically stained area was quantified using ImageJ (v1.8.0).

### 2.6 Transcriptomic analysis

TRIzol reagent (F919KB3054, Sangon Biotech, China) was used to extract total RNA from mouse liver tissues. After assessing purity, quantity, and integrity, transcriptome libraries were constructed and sequenced on the Illumina NovaSeq 6000 platform to generate 150 bp paired-end reads. Raw sequencing data were processed with Fastp to remove low-quality sequences. Differential gene expression analysis was performed using the DESeq2 package in R (v4.3.2), with genes showing |log_2_FC| > 1 and adjusted *p*-value<0.05 considered significantly upregulated or downregulated. Visualization of differential expression results was conducted using the Microbiostatistics platform.

### 2.7 Gene ontology and kyoto encyclopedia of genes and genomes enrichment analysis

GO and KEGG pathway enrichment analyses used a hypergeometric test algorithm. Significantly enriched terms and pathways were identified, and results were visualized using bubble plots (26).

### 2.8 Multiplex immunohistochemistry

Tissue sections were subjected to standard multiplex immunofluorescence staining using the tyramide signal amplification (TSA) method(10534100050, Panovue, China). Briefly, after deparaffinization, rehydration, and heat-induced antigen retrieval, sections were sequentially incubated with primary antibodies against F4/80(GB11027-100, Servicebio, China), iNOS, PKM2, and Arg-1(18985-1-AP, 60268-1-Ig, 66129-1-Ig, Proteintech, China), followed by corresponding secondary antibodies and TSA-conjugated fluorophores.

Between each staining cycle, antibody-TSA complexes were removed by repeating the antigen retrieval step to enable subsequent rounds of staining. Fluorophores were strategically assigned based on target abundance and emission intensity: 690 nm (F4/80), 520 nm (iNOS), 620 nm (PKM2), and 570 nm (Arg-1).

Following completion of all staining cycles, nuclei were counterstained with DAPI, then sections were mounted for later analysis.

### 2.9 Enzyme linked immunosorbent assay

Cytokine levels (IL-1β, IL-6, and IL-10)(88-7013-88, 88-7064-88, 88-7105-88, Invitrogen, USA) were quantified using ELISA kits.

### 2.10 Western blot

Total protein was isolated using RIPA lysis buffer with inhibitors, then concentrations were measured via a BCA assay(WA322434, Thermofisher, USA). SDS-PAGE resolved protein samples, then were electrotransferred to PVDF membranes. The membranes were blocked and primed in a five-minute step before being probed with key target antibodies—GLUT1, GCK, HK2, LDHA (66290-1-Ig, 67216-1-Ig, 66974-1-Ig, 19987-1-AP, Proteintech, China), PKM2, iNOS, and Arg-1—as well as loading controls (GAPDH and Tubulin)(AF2823, AF2839, Beyotime, China). Subsequently, membranes were incubated with HRP-conjugated secondary antibodies. Immunoreactive bands were visualized with an ECL(180-5001, Tanon, China) detection system and quantified by densitometric analysis using ImageJ software.

### 2.11 Real-time quantitative polymerase chain reaction

Total RNA was isolated and reverse-transcribed into cDNA using a Simply P kit (BSC52M1, BioFlux, China). qPCR then used SYBR Green chemistry, with GAPDH and ACTB serving as endogenous reference genes for normalization. The 2^(-ΔΔCt) method was used to determine relative mRNA expression levels, while primer sequences were supplied by Sangon Biotech and Guantai Biotech (Shanghai), as detailed in **Table 1**.

**Table 1:** qRT-PCR primer sequence (5 ‘→3’).

Gene name	Forward(5’→3’)	Reverse(5’→3’)
Gck(M)	GAGATGGATGTGGTGGCAAT	TGGCGGTCTTCATAGTAGCAG
Hk2(M)	GATACGGGGGTCAAAAGAG	GCTTGTTCGGTTGTAACTAGG
Pkm2(M)	CTCTGGGCTTTGCTTCTGTAG	CTTTTGCTGTCTCCTGACTCC
Glut1(M)	CCTGTCCAGACACTTGCCTTC	TCCAGCGGTAAAGCCTCCTA
Ldha(M)	CTTGTGTAGTGGTGACCTGGT	AGTTGGCAGTGTGTCTCAGAG
iNOS(M)	TTGACGCTCGGAACTGTA	GTTGGTGGCATAAAGTATGTG
Arg-1(M)	GCCAGGGACTGACTACCTTAA	AGTTCTGTCTGCTTTGCTGTG
Acss2(M)	TGTGTCAGTTCAGCAATGTTCTCC	CCAGCATAGCCACCACAAGTTC
Pck1(M)	GTGTTTGTAGGAGCAGCCATGAG	GCCGAAGTTGTAGCCGAAGAAG
Pck2(M)	ACCTCTGCTGCCACCAATCC	TTCCCAGTACACACCGCCATC
Pgm2(M)	GAAACCTGAGAAACTATGACGGGAAG	TTGGCTGCTCTTACTGGTA GGAAG
Gapdh(M)	GCTTGGGCTTCCTTTAGGGTA	GATTTCATAACGGCGGTTCATT
GCK(H)	CCTTTCTCGCTGGAATCAATTT	GCCAACAGCTCTGACAGTGTG
HK2(H)	CTATTGGGAGGGATGAGAGTG	GCGGGCTTTCAGATTCAG
PKM2(H)	GTGTTTGCAGCCTGCTCTAGT	TCCTCCTTCTTCCCTTGATTG
GLUT1(H)	CACCACCTCACTCCTGTTACT	CATCCAAACCTCCTACCCT
LDHA(H)	AATAGTTCTGCCACCTCTGAC	TAACACACGGTAAACATCCAC
INOS(H)	GTGCTCTTTGCCTGTATGCTG	TGATTTTCCTGTCTCTGTCGC
ARG-1(H)	CTAGGAATTGGCAAGGTGATG	GTGTGAAAGATGGGTCCAGTC
ACTB (H)	AAGGTGACAGCAGTCGGTT	TGTGTGGACTTGGGAGAGG

### 2.12 Cell lines

The RAW264.7 murine macrophage line and the THP-1 human monocyte line were obtained from the Cell Bank of the Chinese Academy of Sciences. RAW264.7 cells were routinely maintained in complete DMEM at 37 °C in a 5% CO_2_ atmosphere. For experiments, cells were grown at appropriate densities, left to adhere for 24 h, and then switched to low-serum DMEM (1% FBS) containing 100 ng/mL lipopolysaccharide (LPS HY-D1056, MCE, USA) and 50 mM ethanol for 48 h. THP-1 cells were differentiated into macrophage-like cells by culture in complete RPMI-1640 medium containing 100 ng/mL phorbol 12-myristate 13-acetate (PMA ab120297, Abcam, UK) for 48 h. Following differentiation, THP-1 cells received the same stimulation and treatment regimen as RAW264.7 cells. Treatment groups were administered either 200 ng/mL *Gf* extract or 0.5 mM 2-deoxyglucose (2-DG, positive control)(103344-100, Agilent, USA) during the final 24h of stimulation. Post-treatment, cells and culture supernatants were collected for subsequent Western blot/RT-qPCR and ELISA analyses, respectively. (N: control group;M:100ng/ml LPS + 50mM ethanol; Gf: 100ng/ml LPS + 50mM ethanol+200ng/ml *Gf*;2-DG: 100ng/ml LPS + 50mM ethanol+0.5mM 2-DG).

### 2.13 Cell viability assay

The CCK8 (HY–K0301, MCE, USA) was used to evaluate cell viability release, following the manufacturer’s guidelines.

### 2.14 Immunocytochemistry

Following modeling and drug treatments, cells grown on coverslips in 24-well plates were washed with PBS and fixed with 4% paraformaldehyde for 15 min at room temperature. Subsequently, cells were permeabilized with 0.1% Triton X-100 (4°C, 1 min) and immersed in 5% BSA for 60 min. Immunostaining was performed by probing the samples with primary antibodies against PKM2 (1:200) and iNOS (1:50), incubated either at 4 °C overnight. Post PBS washes, the cells were exposed to species-matched fluorescent secondary antibodies (FITC/Cy3, 1:2000)(ab6785, ab6939, Abcam, UK) for 60 min at RT in the dark. Following extensive washing, the nuclei underwent counterstaining with DAPI at a 1:2000 ratio for 15–20 min. The coverslips were eventually washed, affixed to glass slides, and viewed through a laser scanning confocal microscope.

### 2.15 Metabolic flux analysis

Cellular metabolism was profiled with the help of an Agilent Seahorse XFe96 Analyzer. Cells were plated in XF96 microplates and equilibrated for 1 h at 37 °C in unbuffered XF assay medium (pH 7.4) (103575-100) containing 10 mM glucose(103577-100), 1 mM pyruvate(103578-100), and 2 mM glutamine(103579-100), under non-CO_2_ conditions. Glycolytic function(103344-100) was assessed via sequential injection of 10 mM glucose, 1.5 µM oligomycin, and 50 mM 2-deoxyglucose. To evaluate mitochondrial respiration (103015-100), 2 µM oligomycin, 1.5 µM FCCP, and 0.5 µM antimycin A/rotenone were added one after the other. Metabolic parameters were automatically recorded and normalized using integrated Seahorse Software and a Cytation 7 imaging system. All reagents and kits were obtained from Agilent Technologies (USA).

### 2.16 Statistical examination

Results are shown as mean ± standard deviation, and statistical analysis was conducted with SPSS 27.0. A one-way ANOVA with Tukey’s *post hoc* test was utilized for multiple group comparisons, provided the data adhered to normality and variance homogeneity assumptions. For data violating these assumptions, Dunn’s *post hoc* analysis was applied after the Kruskal-Wallis test. For two-group comparisons, the Mann-Whitney U test was utilized, contingent on parametric test assumptions. Statistical significance was assigned to p-values below 0.05. GraphPad Prism 10 was used for all graphical displays.

## 3 Results

### 3.1 Qualitative and quantitative analysis of the *Gf* extract

Comprehensive chemical analysis revealed that *Gf* extract contains diverse bioactive components, including alkaloids, flavonoids, carotenoids, terpenoids, sterols, saponins, polysaccharides, amino acids, and essential minerals (selenium, potassium, cobalt). Additionally, microbial components (A, B, C, D, E, and P) were detected. Seven representative bioactive compounds—ascorbic acid, gallic acid, vanillic acid, caffeic acid, p-coumaric acid, ferulic acid, and cinnamic acid—were selected as reference standards based on literature.

**Figure 1:**
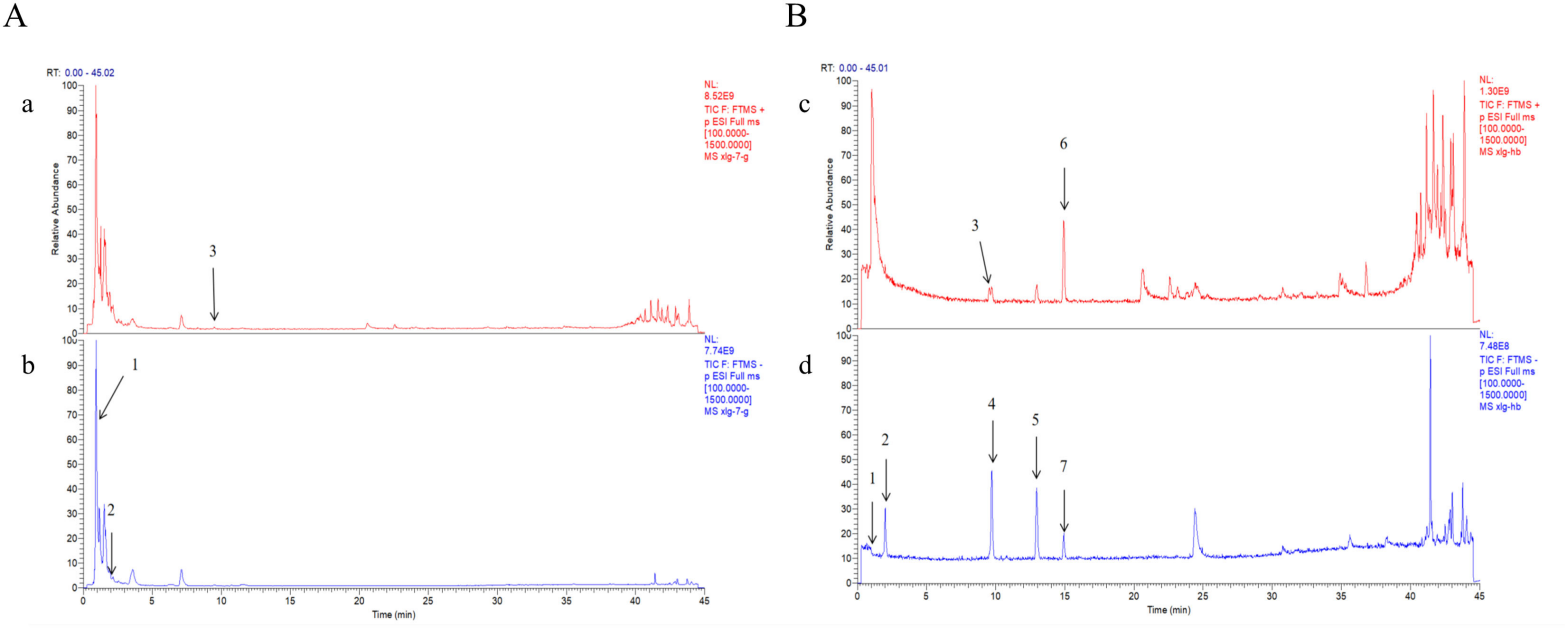
Identification of representative components in *Gf* extract. **(A)** UHPLC-Q-Orbitrap HRMS total ion chromatogram of the *Gf* extract (a: positive ion mode; b: negative ion mode). **(B)** UHPLC-Q-Orbitrap HRMS total ion chromatogram of the *Gf* extract mixed standard solution (c: positive ion mode; d: negative ion mode). Peaks: 1) Ascorbic acid; 2) Gallic acid; 3) Vanillic acid; 4) Caffeic acid; 5) p-Coumaric acid; 6) Ferulic acid; 7) Cinnamic acid.

The chemical characterization of these compounds was performed using UHPLC-Q-Exactive Orbitrap HRMS. Total ion chromatograms (TIC) of the extract and the mixed standards are shown in **Figure 1**, with detailed information on retention times, molecular weights, and fragment ions summarized in **Table 2**.

**Table 2:** *Gf* represents basic information on chemical composition.

Number	Name	Molecular formula	Mode	Molecular weight	RT value (min)	Peak area(10^6)		content(μg/g)
						Mixed standard	*Gf*	*Gf*
1	Ascorbic acid	C6H8O6	[M-H]-	175.0237	1.11	21.54	6.80	7.89
2	Gallic acid	C7H6O5	[M-H]-	169.0131	2.01	709.22	7.57	0.27
3	Vanillic acid	C8H8O4	[M+H]+	169.0495	9.54	44.37	0.17	0.10
4	Caffeic acid	C9H8O4	[M-H]-	179.0339	9.71	1359.19	0.00	0.00
5	*para*-coumaric acid	C9H8O3	[M-H]-	163.0390	12.97	1068.42	0.00	0.00
6	Ferulic acid	C10H10O4	[M+H]+	195.0652	14.93	585.47	0.00	0.00
7	Cinnamic acid	C9H8O2	[M+HCOO]-	193.0495	14.94	296.53	0.00	0.00

### 3.2 *Gf* extract ameliorates alcoholic liver disease

**Figure 2:**
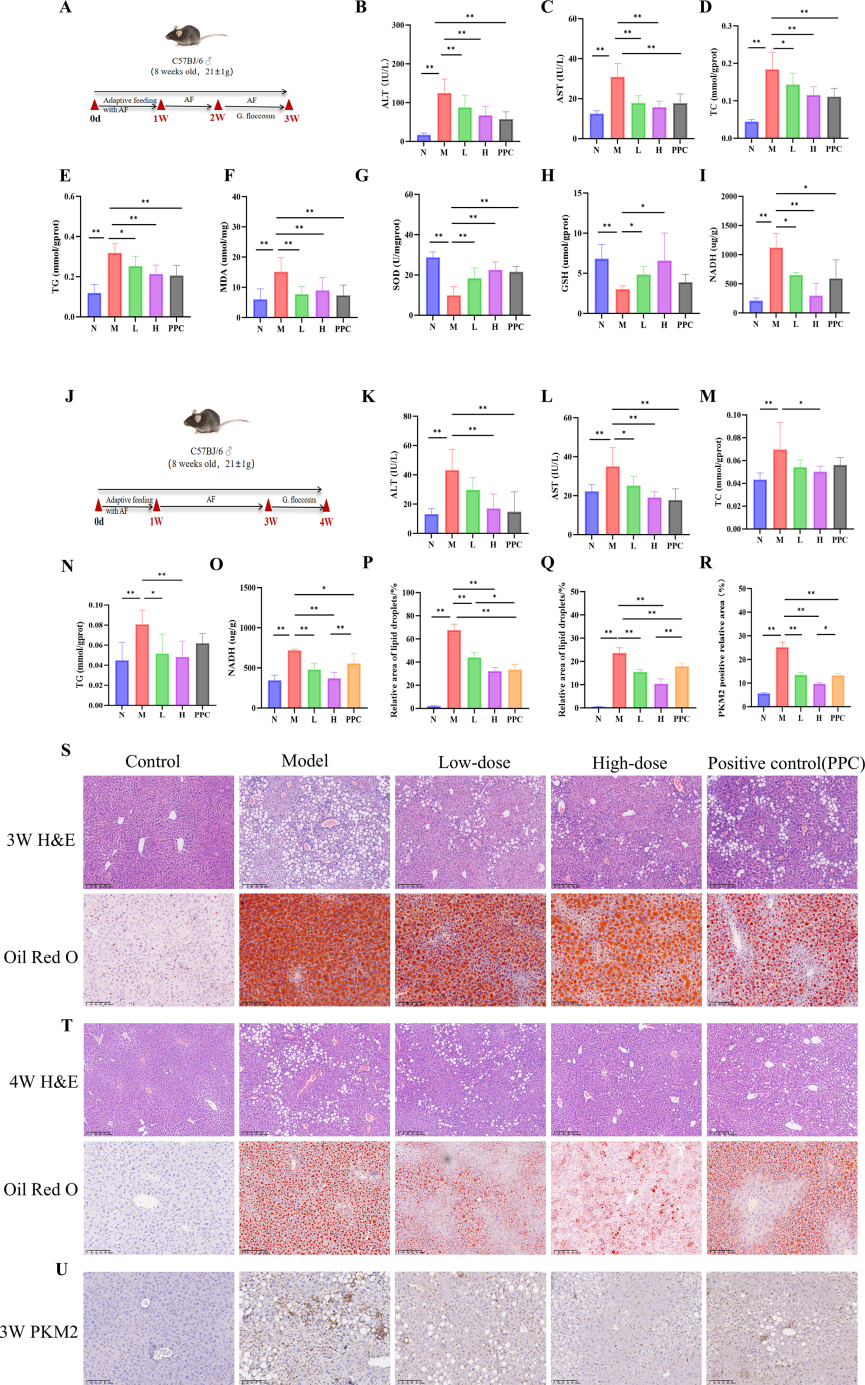
*Gf* Extract ameliorates alcoholic liver disease in mice. **(A)** Experimental timeline of the 3-week protoco. Group sizes: Normal (N), n = 8; Model (M) and Low-dose *Gf* extract (L), n = 12; High-dose *Gf* extract (H) and Polyene Phosphatidylcholine Capsules (PPC), n = 11. **(B, C)** Serum levels of alanine aminotransferase (ALT) and aspartate aminotransferase (AST). **(D–I)** Hepatic levels of total cholesterol (TC), triglycerides (TG), malondialdehyde (MDA), total superoxide dismutase (T-SOD), glutathione (GSH), and nicotinamide adenine dinucleotide (NADH) in tissue homogenate supernatant. **(J)** Experimental timeline of the 4-week protocol. All groups, n = 8. **(K, L)** Serum ALT and AST levels. **(M–O)** Hepatic levels of TC, TG, and NADH in tissue homogenate supernatant. **(P)** Quantitative analysis of Oil Red O staining (3-week groups). **(Q)** Quantitative analysis of Oil Red O staining (4-week groups). **(R)** Quantitative analysis of the relative PKM2-positive area from immunohistochemistry. **(S)** Representative hematoxylin and eosin (H&E) staining (100×, scale bar = 200 μm) and Oil Red O staining (200×, scale bar = 100 μm) of liver sections from the 3-week groups. **(T)** Representative H&E staining (100×, scale bar = 200 μm) and Oil Red O staining (200×, scale bar = 100 μm) of liver sections from the 4-week groups. **(U)** Representative immunohistochemical staining of PKM2 in liver tissue from the 3-week group. Data are presented as mean ± SD. *p < 0.05, **p < 0.01. AF, Alcohol-fed; N, Normal control group; M, Model group; L, AF with low-dose Gf extract (0.628 g/kg) group; H, AF with high-dose Gf extract (1.255g/kg) group; PPC, Polyene Phosphatidylcholine Capsules.

The ALD model and its treatment protocol are shown in **Figure 2**. In the 3-week model, mice were subjected to one week of adaptive feeding with an alcohol-containing diet, followed by one week of modeling during which drug treatment was administered concurrently (**Figure 2A**). Evaluation of this model revealed significantly elevated serum ALT and AST levels in the alcohol-fed group (**Figures 2B, C**), along with increased tissue TC and TG contents, heightened oxidative stress markers (MDA and NADH), and decreased T-SOD and GSH activities (**Figures 2D–I**), confirming successful induction of alcoholic liver disease. After one week of treatment with *Gf* extract, improvements were observed in liver function, lipid metabolism, and oxidative stress markers, with significant reductions in MDA and NADH levels and increased T-SOD and GSH activities. The high-dose *Gf* group demonstrated superior efficacy compared to the low-dose group, and it significantly outperformed the positive control drug Polyene Phosphatidylcholine in lowering NADH levels.

In the 4-week model, adaptive feeding was followed by two weeks of alcohol-induced liver injury, with drug treatment administered during the final week after modeling cessation (**Figure 2J**). Although the alcohol-fed group exhibited elevated serum and tissue indices similar to those in the 3-week model, the increases were less pronounced. Drug administration ameliorated all measured parameters, with the high-dose *Gf* group again showing stronger effects compared to the low-dose group (**Figures 2K–O**).

Histopathological analysis supported these findings. Liver tissues from alcohol-fed mice showed macrovesicular and microvesicular steatosis, ballooning degeneration, and neutrophil infiltration, while Oil Red O staining confirmed substantial lipid droplet accumulation in hepatocytes. Pathological injury was less severe in the 4-week model compared to the 3-week model. Treatment with *Gf* extract markedly alleviated hepatic steatosis, ballooning degeneration, and inflammatory cell infiltration, with the high-dose group exhibiting more substantial improvement than the low-dose group (**Figures 2P–Q, S, T**).

These results collectively demonstrate that *Gf* extract effectively ameliorates alcoholic liver disease, particularly at high doses, by improving liver function, reducing lipid accumulation, and mitigating oxidative stress and histopathological damage.

### 3.3 Transcriptomic analysis identifies a glycolysis-immune regulation hub in alcoholic liver disease

**Figure 3:**
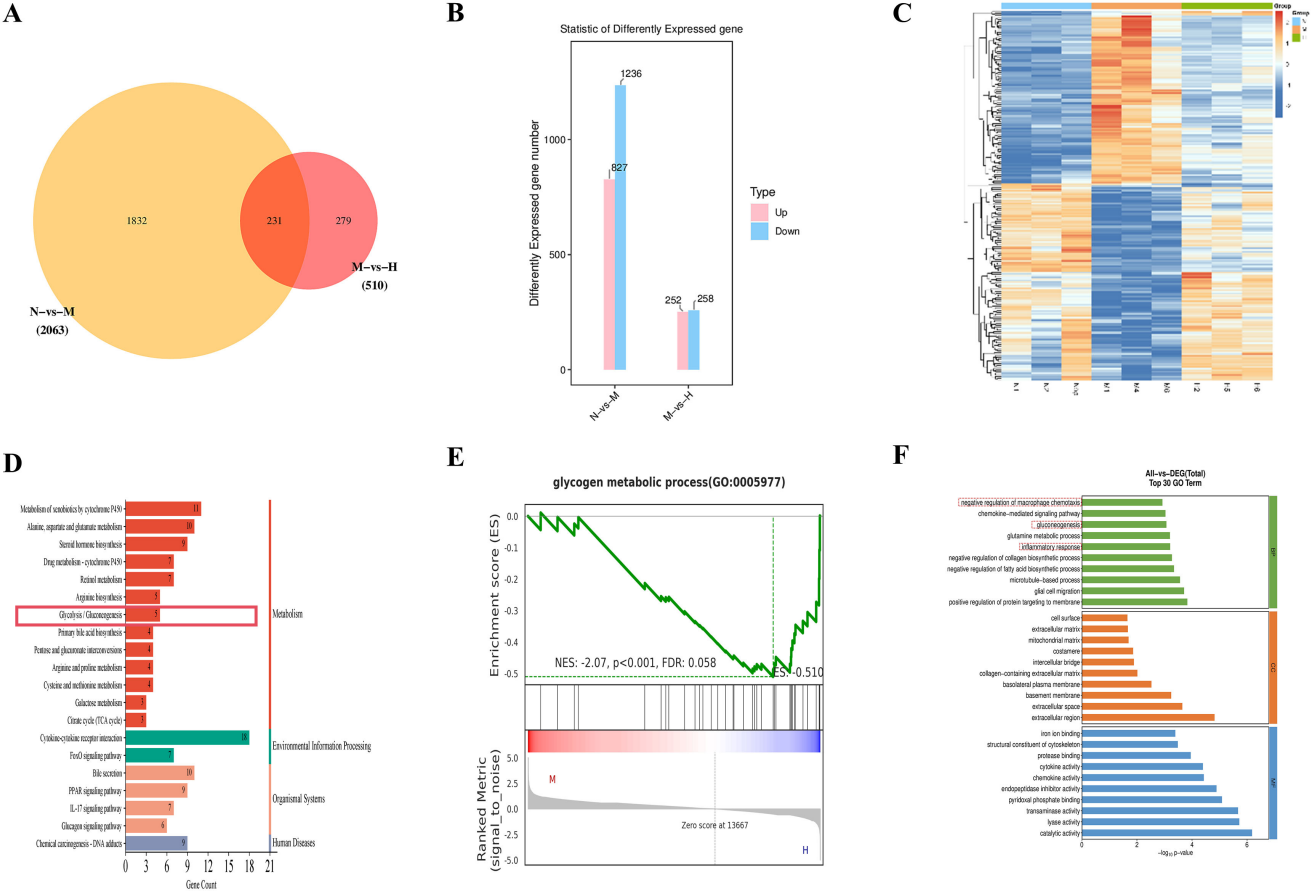
Transcriptomic analysis identifies a glycolysis–immune regulation hub in alcoholic liver disease. **(A)** Venn diagram. **(B)** Statistics of differentially expressed genes. **(C)** Heatmap. **(D)** KEGG pathway analysis. **(E)** GSEA plot for Glycogen Metabolic Process **(F)** GO enrichment analysis.

Transcriptome sequencing was performed on liver tissues from the alcohol-fed control (N), alcohol-fed model (M), and high-dose *Gf* extract-treated (H) groups. Venn diagram analysis identified 231 overlapping differentially expressed genes (DEGs) between the N vs. M and M vs. H comparisons (**Figure 3A**). Compared to the H group, the M group exhibited the upregulation of 252 genes and the downregulation of 258 genes (**Figure 3B**). The expression profiles of these DEGs across the N, M, and H groups are visualized in a heatmap (**Figure 3C**).

KEGG enrichment analysis of the 231 overlapping DEGs revealed that the top 20 enriched pathways included glycolysis/gluconeogenesis (**Figure 3D**). Key genes implicated in this pathway, such as *Gck*, *Acss2*, *Pck1*, *Pck2*, and *Pgm2*, were differentially expressed. Gene set enrichment analysis (GSEA) further confirmed the broad impact of *Gf* extract on hepatic metabolism. Although the glycolysis gene set did not reach statistical significance, significant enrichment was observed in the ‘glycogen metabolic process’ (**Figure 3E**). This finding suggests that *Gf* extract orchestrates a comprehensive reprogramming of carbohydrate metabolism in the liver. Specifically, it appears to attenuate glycolytic flux while modulating glycogen-related energy storage, thereby contributing to the restoration of metabolic homeostasis in alcoholic liver disease (ALD).

GO annotation further revealed that these DEGs were associated with biological processes (BP), cellular components (CC), and molecular functions (MF). Among the top 10 enriched BP terms were “negative regulation of macrophage chemotaxis,” “gluconeogenesis,” and “inflammatory response” (**Figure 3F**).

Immunohistochemical staining demonstrated prominent PKM2 expression localized to hepatic sinusoids (**Figures 2R, U**). Previous studies have established that macrophages undergo significant metabolic reprogramming during immune activation, with glycolysis serving as a core metabolic hallmark of their pro-inflammatory phenotype. Based on these findings, we hypothesize that *Gf* extract mitigates alcohol-induced liver injury by modulating glycolysis in macrophages, thereby attenuating the inflammatory response and restoring hepatic metabolic balance.

### 3.4 *Gf* extract inhibits M1 pro-inflammatory macrophage polarization

**Figure 4:**
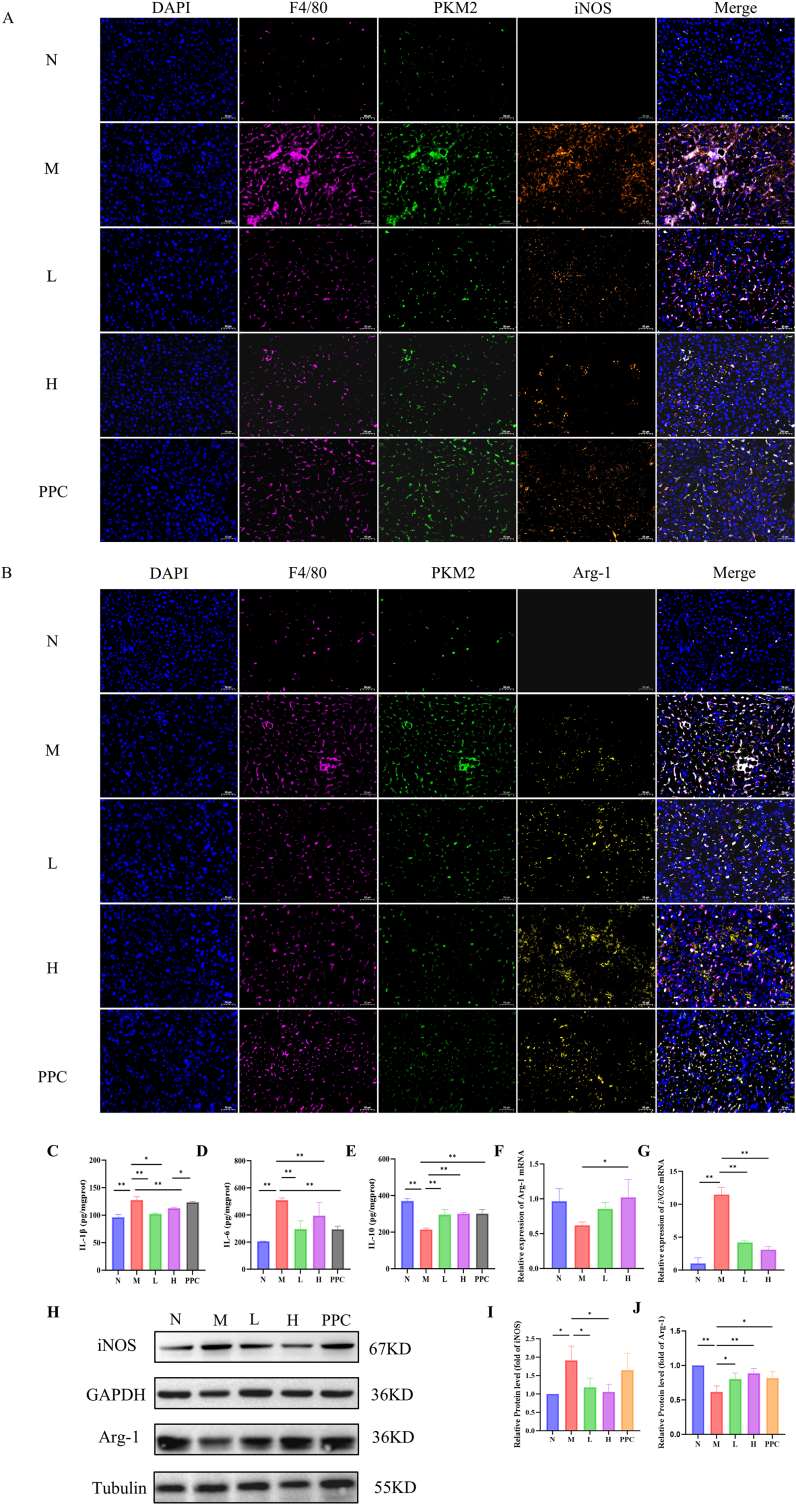
*Gf* Extract inhibits M1 pro-inflammatory macrophage polarization. **(A)** Immunofluorescence histochemistry staining of F4/80, PKM2 and iNOS in liver tissue sections. **(B)** Immunofluorescence histochemistry staining of F4/80, PKM2 and Arg-1 in liver tissue sections (400×, scale bar = 50 μm). **(C-E)** The levels of IL-1β, IL-6 and IL-10 in liver tissues were detected by ELISA. **(F, G)** The relative mRNA expression of iNOS and Arg-1 in liver tissues were detected by RT-qPCR (n = 5). **(H)** The protein expression of iNOS and Arg-1 in liver tissues were detected by western blot (n = 3). **(I, J)** Quantitative assessment of iNOS and Arg-1 protein levels (n = 3). Data are presented as mean ± SD. *p < 0.05, **p < 0.01.

Multiplex immunohistochemical analysis demonstrated that *Gf* extract reversed the polarization imbalance of hepatic macrophages in a dose-dependent manner. Specifically, treatment with *Gf* extract significantly reduced the number of F4/80+PKM2+iNOS+ M1 macrophages while restoring the population of F4/80+PKM2+Arg-1+ M2 macrophages in the livers of the M group (**Figures 4A, B**). Consistent with these findings, Western blotting and qPCR analyses further confirmed that *Gf* extract downregulated the expression of the M1 polarization marker iNOS while upregulating the expression of the M2 polarization marker Arg-1 at both the protein and transcriptional levels (**Figures 4F–J**).

This phenotypic shift in macrophage polarization was accompanied by a reduction in the inflammatory response, as evidenced by decreased levels of pro-inflammatory cytokines and increased levels of anti-inflammatory cytokines (**Figures 4C–E**). These results collectively suggest that *Gf* extract exerts its anti-inflammatory effects by modulating macrophage polarization, specifically by suppressing the M1 pro-inflammatory phenotype and promoting the M2 anti-inflammatory phenotype, thereby contributing to the resolution of liver inflammation in alcoholic liver disease.

### 3.5 *Gf* extract regulates the expression of key glycolytic enzymes and metabolites

**Figure 5:**
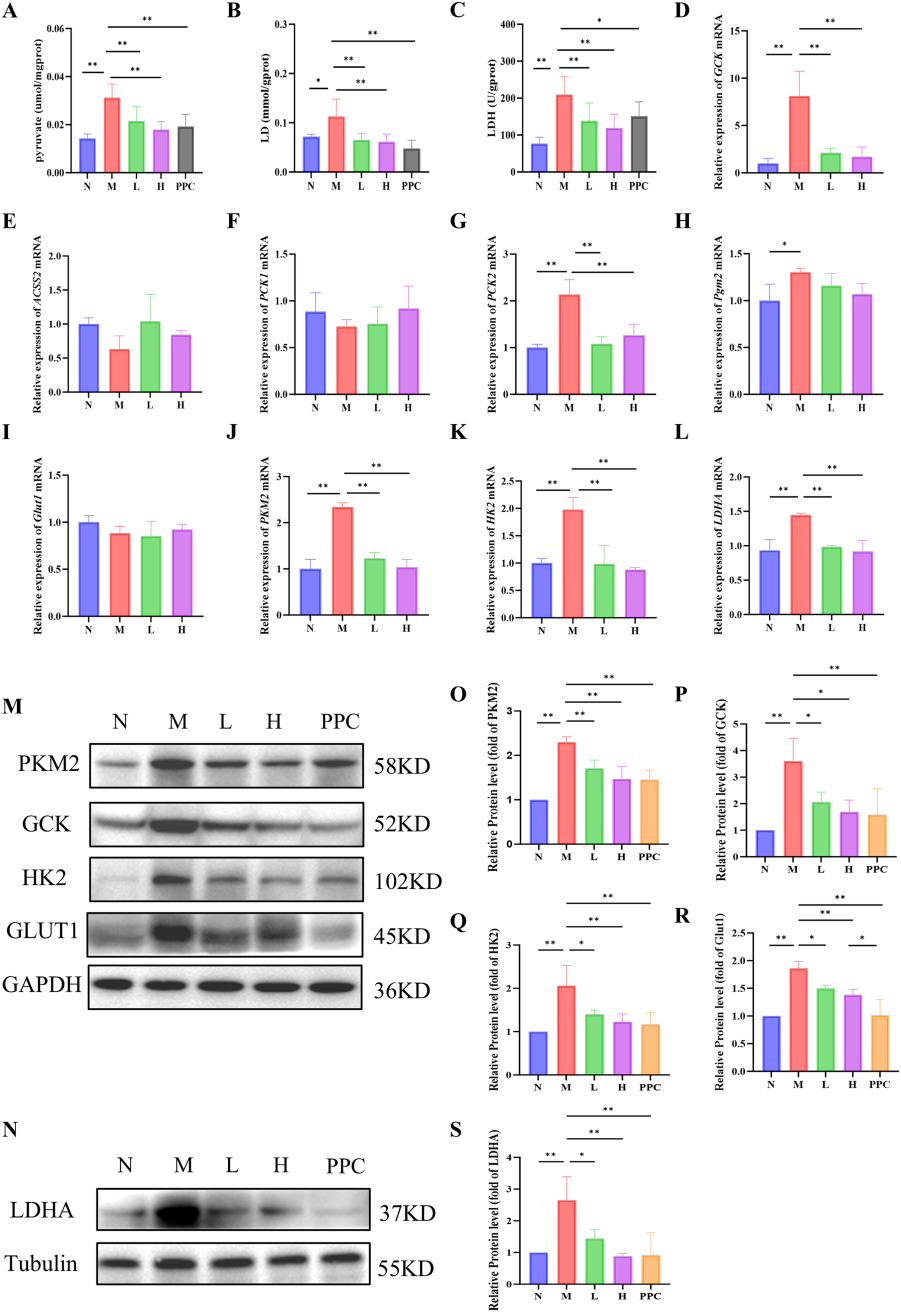
*Gf* extract regulates the expression of key glycolytic enzymes and metabolites. **(A-C)** The contents of pyruvate, lactate (LD) and lactate dehydrogenase (LDH) in liver tissues. **(D-L)** The relative mRNA expression of *Gck*, *Acss2*, *Pck1*, *Pck2*, *Pgm2*, *Glut1*, *Pkm2*, *Hk2* and *Ldha* in liver tissues were detected by RT-qPCR (n = 5). **(M, N)** The protein expression of PKM2, GCK, HK2, GLUT1 and LDHA in liver tissues were detected by western blot (n = 3). **(O-S)** Quantitative assessment of PKM2, GCK, HK2, GLUT1 and LDHA protein levels (n = 3). Data are presented as mean ± SD. *p < 0.05, **p < 0.01.

Metabolite analysis revealed that the levels of pyruvate, lactate, and lactate dehydrogenase (LDH) were significantly elevated in the M group compared to the N group. Treatment with *Gf* extract markedly reduced these levels, indicating a potential regulatory effect on glycolytic activity (**Figures 5A–C**).

To further investigate the molecular mechanisms underlying these metabolic changes, we analyzed the expression of key genes involved in the KEGG glycolysis/gluconeogenesis pathway, including *Gck*, *Acss2*, *Pck1*, *Pck2*, and *Pgm2*. Among these, only *Gck* and *Pck2* exhibited significant intergroup differences (**Figures 5D–H**). Notably, *Gck* mRNA expression displayed the most pronounced upregulation in the model group, highlighting its role as a pivotal regulator of glycolysis initiation. This prompted further investigation into the expression of other key glycolytic enzymes, including *Glut1*, *Pkm2*, *Hk2*, and *Ldha*.

While no significant differences in *Glut1* mRNA expression were observed among the groups (**Figure 5I**), Western blot analysis demonstrated a significant reduction in the protein levels of GLUT1, PKM2, HK2, and LDHA following *Gf* extract treatment (**Figures 5M–S**). Consistently, the mRNA levels of *Pkm2*, *Hk2*, and *Ldha* were also significantly downregulated in the treatment groups (**Figures 5J–L**).

These findings suggest that *Gf* extract exerts its therapeutic effects on alcoholic liver disease by modulating glycolytic enzyme expression and reducing the accumulation of glycolytic metabolites, thereby contributing to the restoration of metabolic homeostasis.

### 3.6 *Gf* extract suppresses M1 macrophage polarization via downregulating PKM2/iNOS in RAW264.7 cells

**Figure 6:**
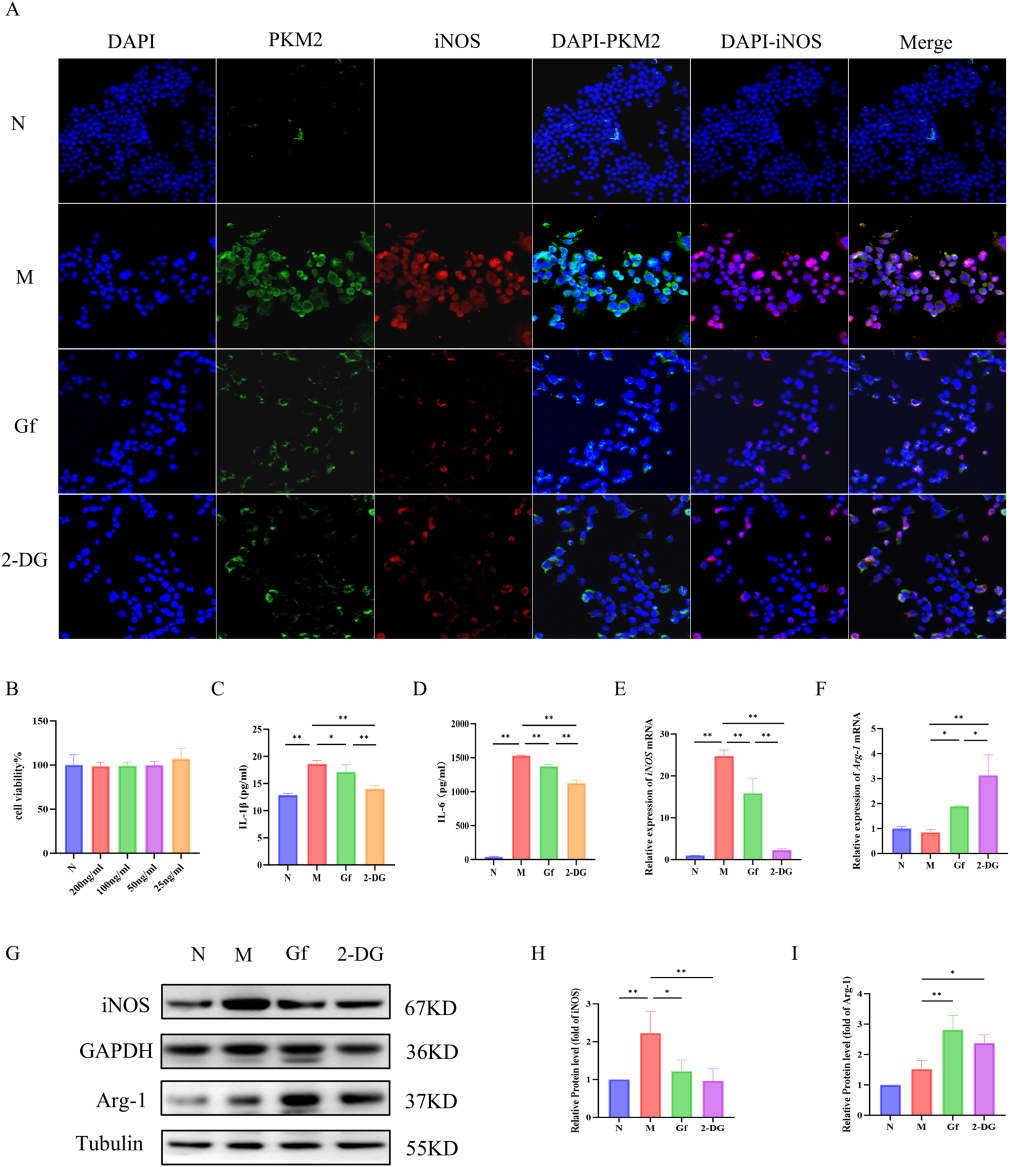
*Gf* extract suppresses M1 macrophage polarization via downregulating PKM2/iNOS in RAW264.7 cells. **(A)** Cellular immunofluorescence staining of PKM2 and iNOS in murine macrophage RAW264.7 cells (400×, scale bar = 50 μm). **(B)** Cytotoxicity assessment of *Gf* extract. **(C, D)** The levels of IL-1β and IL-6 in RAW264.7 cells were detected by ELISA. **(E, F)** The relative mRNA expression of iNOS and Arg-1 in RAW264.7 cells were detected by RT-qPCR (n = 5). **(G)** The protein expression of iNOS and Arg-1 in RAW264.7 cells were detected by western blot (n = 3). **(H, I)** Quantitative assessment of iNOS and Arg-1 protein levels (n = 3). Data are presented as mean ± SD. *p < 0.05, **p < 0.01. Gf group: Cells were stimulated with 100 ng/mL LPS plus 50 mM ethanol and cotreated with 200 ng/mL Gf extract; 2-DG group: Cells were stimulated with 100 ng/mL LPS plus 50 mM ethanol and co-treated with 0.5 mM 2-DG.

The CCK-8 assay identified 200 ng/mL as the optimal non-cytotoxic concentration of the *Gf* extract for *in vitro* experiments (**Figure 6B**). An *in vitro* M1 macrophage polarization model was established by treating RAW264.7 cells with 100 ng/mL LPS and 50 mM ethanol for 48 hours, followed by 24-hour co-incubation with the *Gf* extract.

Immunofluorescence co-staining showed increased PKM2 and iNOS expression in the model group, which was significantly suppressed by *Gf* extract treatment (**Figure 6A**). Similarly, ELISA results revealed elevated IL-1β and IL-6 levels in the model group, which were markedly reduced after treatment (**Figures 6C, D**). Western blotting and qPCR further confirmed that *Gf* extract downregulated iNOS expression at both mRNA and protein levels, while upregulating the M2 marker Arg-1 (**Figures 6E–I**).

These results suggest that *Gf* extract alleviates inflammation by suppressing M1 polarization through the downregulation of PKM2 and iNOS and promoting M2 polarization via Arg-1 upregulation.

### 3.7 *Gf* extract reprograms metabolism in RAW264.7 macrophages by suppressing glycolysis and enhancing oxidative phosphorylation

**Figure 7:**
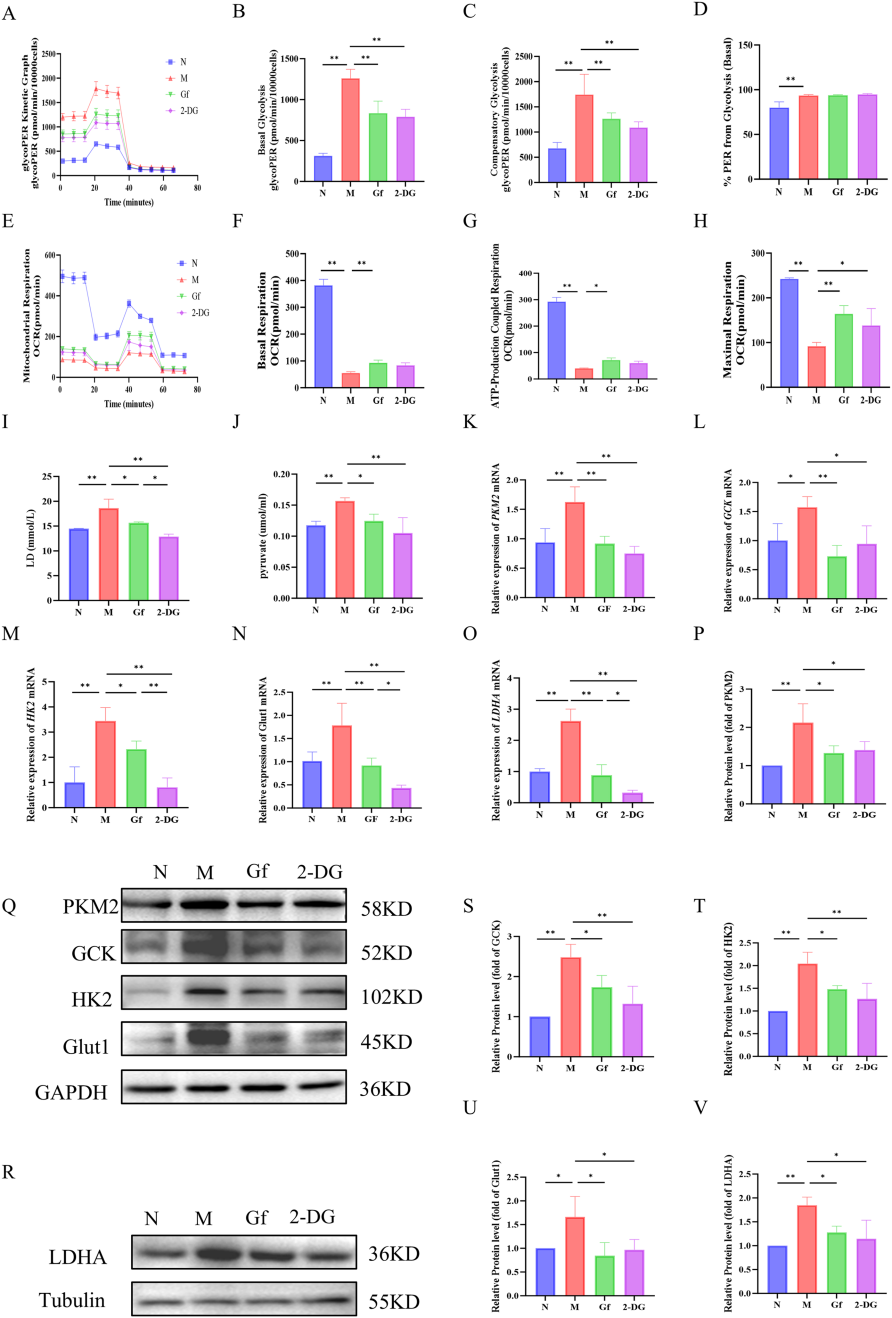
*Gf* extract reprograms metabolism in RAW264.7 macrophages by suppressing glycolysis and enhancing oxidative phosphorylation. **(A-D)** Glycolytic kinetic parameters in RAW264.7 cells: glycoPER Kinetic Graph, basal and compensatory glycolysis levels, and the % PER from Glycolysis (Basal) (n = 8). **(E-H)** Mitochondrial respiratory parameters: basal respiration, ATP-Production coupled respiration, and maximal respiration (n = 8). **(I, J)** The content of lactate (LD) and pyruvate in the cell culture supernatant (n = 8). **(K-O)** The relative mRNA expression of *Pkm2*, *Gck*, *Hk2*, *Glut1*, and *Ldha* in RAW264.7 cells were detected by RT-qPCR (n = 5). **(Q, R, P, S-V)** The protein levels of PKM2, GCK, HK2, GLUT1, and LDHA were detected by Western blot and quantitative analysis (n = 3). Data are presented as mean ± SD. *p < 0.05, **p < 0.01.

To explore the metabolic reprogramming induced by *Gf* extract in RAW264.7 macrophages, we first assessed glycolytic function. The model group exhibited significantly elevated glycolytic rates, including basal and compensatory glycolysis, all of which were markedly suppressed by *Gf* extract, comparable to the effect of the glycolytic inhibitor 2-DG (**Figures 7A–D**). Despite these changes, the proportional contribution of glycolysis to basal energy production remained stable, suggesting that *Gf* extract reduces overall glycolytic capacity without altering energy source allocation.

Mitochondrial respiration analysis revealed severe dysfunction in the model group, with decreased basal respiration, ATP-linked respiration, and maximal respiratory capacity. *Gf* extract significantly restored these parameters, outperforming 2-DG, which had no notable effect on mitochondrial function (**Figures 7E–H**).

At the molecular level, the model group showed increased lactate and pyruvate accumulation, along with upregulation of glycolytic enzymes (*Pkm2*, *Gck*, *Hk2*, *Glut1*, and *Ldha*) at both mRNA and protein levels. These alterations were effectively reversed by *Gf* extract treatment (**Figures 7I–V**).

In summary, *Gf* extract reprograms macrophage metabolism by suppressing glycolysis and enhancing mitochondrial oxidative phosphorylation, restoring metabolic homeostasis in M1-polarized macrophages.

### 3.8 *Gf* extract suppresses M1 polarization in human macrophages by targeting the PKM2/iNOS axis

**Figure 8:**
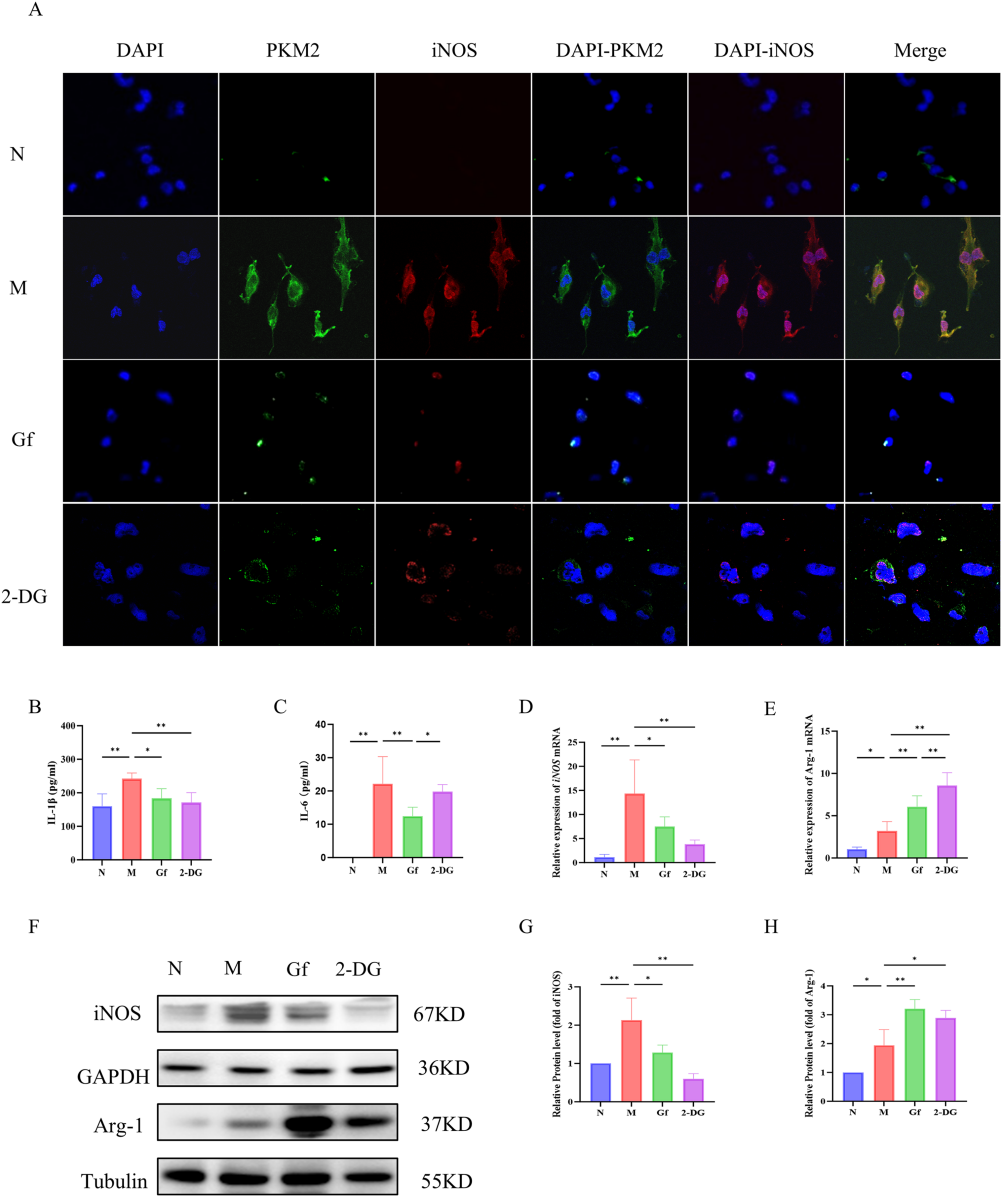
*Gf* extract suppresses M1 polarization in human macrophages by targeting the PKM2/iNOS axis. **(A)** Cellular immunofluorescence staining of PKM2 and iNOS in THP-1 cells. **(B, C)** The levels of IL-1β and IL-6 in THP-1 cells were detected by ELISA. **(D, E)** The relative mRNA expression of *iNOS* and *Arg-1* in THP-1 cells were detected by RT-qPCR (n = 5). **(F-H)** The protein levels of iNOS and Arg-1 were detected by Western blot and quantitative analysis (n = 3). Data are presented as mean ± SD. *p < 0.05, **p < 0.01.

To evaluate the cross-species relevance of *Gf* extract, its effects were tested in PMA-differentiated human THP-1 macrophages. Similar to murine cells, *Gf* extract downregulated PKM2 and iNOS protein expression (**Figure 8A**) and significantly decreased extracellular lactate and pyruvate levels (**Figures 8B, C**). Additionally, the extract suppressed iNOS expression while enhancing Arg-1 at both mRNA and protein levels (**Figures 8D–H**), indicating effective inhibition of M1 polarization.

These findings confirm that the macrophage-polarizing effects of *Gf* extract are conserved between murine and human macrophages.

### 3.9 *Gf* extract reprograms metabolism in human THP-1 macrophages by suppressing glycolysis and enhancing oxidative phosphorylation

**Figure 9:**
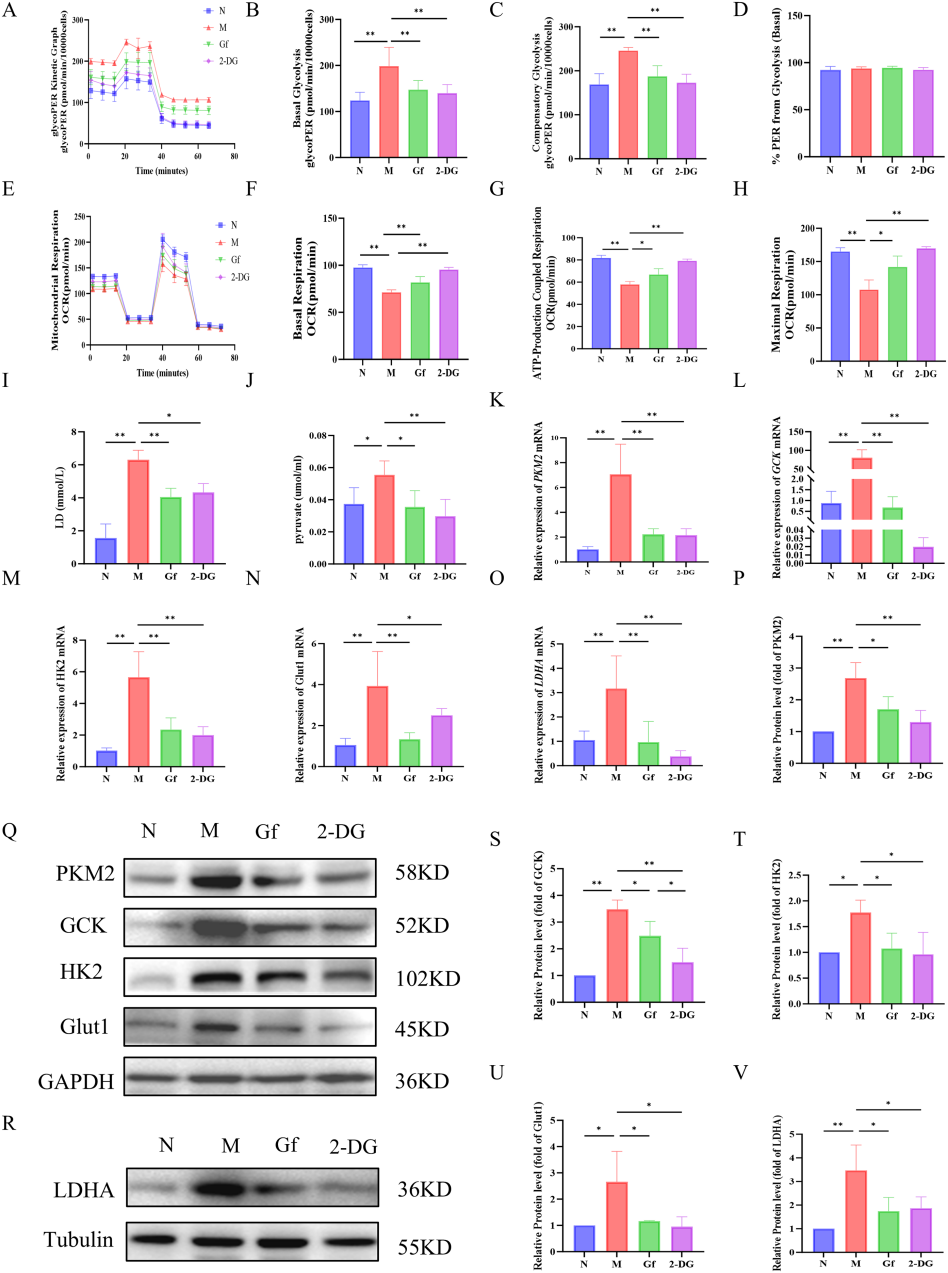
*Gf* extract reprograms metabolism in human THP-1 macrophages by suppressing glycolysis and enhancing oxidative phosphorylation. **(A-D)** Glycolytic kinetic parameters in THP-1 cells: glycoPER Kinetic Graph, basal and compensatory glycolysis levels, and the % PER from Glycolysis (Basal) (n = 8). **(E-H)** Mitochondrial respiratory parameters: basal respiration, ATP-Production coupled respiration, and maximal respiration (n = 8). **(I, J)** The content of lactate (LD) and pyruvate in the cell culture supernatant (n = 8). **(K-O)** The relative mRNA expression of *Pkm2*, *Gck*, *Hk2*, *Glut1*, and *Ldha* in THP-1 cells were detected by RT-qPCR (n = 5). **(Q, R, P, S-V)** The protein levels of PKM2, GCK, HK2, GLUT1, and LDHA were detected by Western blot and quantitative analysis (n = 3). Data are presented as mean ± SD. *p < 0.05, **p < 0.01.

To determine whether *Gf* extract induces similar metabolic reprogramming in human macrophages, its effects were examined in PMA-differentiated THP-1 cells. *Gf* extract significantly suppressed glycolytic flux, including basal and compensatory glycolysis (**Figures 9A–D**).

Additionally, *Gf* extract restored mitochondrial respiratory function, improving basal respiration, ATP production, and maximal respiratory capacity, comparable to the effects of the glycolytic inhibitor 2-DG (**Figures 9E–H**).

At the molecular level, *Gf* extract reversed the accumulation of lactate and pyruvate in the supernatant and downregulated the expression of key glycolytic genes (*Pkm2*, *Gck*, *Hk2*, *Glut1*, and *Ldha*) and their protein products. Notably, the 50-fold upregulation of Gck observed in the model group was normalized by *Gf* extract treatment (**Figures 9I–V**).

## 4 Discussion

Alcoholic liver disease (ALD) poses a significant global health burden, with no approved targeted therapies to effectively halt disease progression. Thus, alcohol abstinence remains the most effective strategy for improving outcomes across all stages of the disease (27). This highlights the urgent need for novel therapeutic approaches targeting the underlying pathogenesis of ALD. Natural products derived from medicinal fungi, characterized by their multi-target actions and low toxicity, represent a promising source of candidate agents (28). *Gf*, a traditional remedy for hepatobiliary disorders, has long been underutilized due to undefined active constituents. To address these limitations and validate its therapeutic potential, this study established standardized quality control for *Gf* extract and systematically demonstrated, for the first time, its efficacy in ALD via regulation of the macrophage glycolysis-M1 polarization axis.

Using UHPLC-Q-Exactive Orbitrap HRMS, we identified ascorbic acid, gallic acid, and vanillic acid as the primary bioactive components of *Gf* extract. Beyond their reported pharmacological profiles (29–34), these compounds likely act synergistically to regulate macrophage immunometabolism. Ascorbic acid, a potent antioxidant and cofactor for prolyl hydroxylases, may suppress the LPS-induced glycolytic switch in macrophages by promoting HIF-1α degradation (35). It also activates PPARα, which enhances fatty acid oxidation, ameliorating hepatic steatosis and reducing lipid-driven macrophage inflammation (36). Gallic acid, through AMPK activation, inhibits glycolysis and promotes oxidative phosphorylation, favoring M2 polarization (36–38, 41). Additionally, its antioxidant properties and modulation of CYP enzymes may mitigate alcohol-induced hepatocyte injury, reducing macrophage recruitment (37, 41). Vanillic acid, through gut microbiota modulation and anti-fibrotic effects, may lower systemic inflammation and reduce macrophage activation (39, 40). Together, these components directly and indirectly target the macrophage glycolysis-M1 axis, forming the chemical basis for *Gf*’s multi-target therapeutic effects in ALD.

The hepatoprotective effects of *Gf* extract were demonstrated in both preventive and therapeutic ALD models [Patent Application No.2025114372258]. In the preventive model, *Gf* extract significantly improved liver function, reduced hepatic lipid accumulation, and mitigated oxidative stress and inflammation, consistent with findings from other herbal extracts in ALD (41–43). However, in the therapeutic model, despite limited effects on systemic oxidative stress markers, *Gf* extract improved liver function and lipid metabolism. This suggests that its efficacy in advanced ALD stems from mechanisms beyond direct antioxidant activity, likely through targeting immune-driven pathology. The differential impact of *Gf* extract between the two models highlights the importance of disease stage, as the therapeutic model reflects a condition dominated by sustained immune cell dysregulation rather than acute oxidative stress. These findings align with our central observation that *Gf* extract regulates macrophage glycolysis and polarization, which are critical for driving inflammation in established ALD (44).

Transcriptomic analysis provided critical insights into the therapeutic mechanism of *Gf* extract. KEGG and GO analyses identified a core pathological process underlying ALD: the interplay between glycolytic reprogramming and immune inflammation (45). Notably, the significant suppression of the “Glycolysis” pathway, alongside the concurrent “negative regulation of macrophage chemotaxis,” strongly indicates that *Gf* extract exerts its therapeutic effects by remodeling the hepatic immune-metabolic microenvironment (28). These findings align with the emerging paradigm of targeting macrophage glycolysis as a potential therapeutic strategy in ALD (19, 46).

At the mechanistic level, experimental data revealed that *Gf* extract coordinately downregulates multiple key rate-limiting enzymes in the glycolytic pathway. Chronic alcohol exposure disrupts hepatic immune homeostasis by promoting glycolytic reprogramming in macrophages: ethanol not only enhances the expression of glycolytic key enzymes (e.g., GCK, HK2) but also induces PKM2 nuclear translocation, thereby facilitating M1 polarization and pro-inflammatory cytokine secretion. This alcohol-driven metabolic switch in macrophages represents a critical pathological link in ALD progression. Specifically, it inhibited GLUT1, the primary glucose transporter, whose upregulation is recognized as a critical early event driving M1 polarization (47–49). Additionally, *Gf* extract significantly suppressed GCK, the enzyme responsible for catalyzing the first step of glycolysis. In the THP-1 model, stimulation with LPS and ethanol triggered a dramatic upregulation of Gck mRNA expression (by tens of folds), an effect that was effectively reversed by *Gf* extract. This observation corroborates recent studies highlighting the LRH-1/GCK axis as a key regulator of macrophage inflammatory responses (50). Furthermore, *Gf* extract downregulated HK2 expression, another pivotal glycolytic enzyme. Previous studies have demonstrated that HK2 transcriptional inhibition reduces glycolysis and pro-inflammatory cytokine secretion in macrophages (51), while its overexpression in MASLD models has been shown to drive a pro-inflammatory feedback loop (52).

Among these glycolytic enzymes, PKM2 emerges as a particularly critical target. Immunofluorescence analysis revealed that in macrophages exposed to LPS and ethanol, PKM2 translocated to the nucleus, where it acts as a transcriptional coactivator of HIF-1α, enhancing the expression of M1 polarization markers such as iNOS (53–55). Treatment with *Gf* extract significantly inhibited PKM2 expression and nuclear translocation, thereby reducing iNOS levels and the downstream secretion of pro-inflammatory cytokines IL-1β and IL-6. This suggests that *Gf* extract disrupts both the metabolic function of cytoplasmic PKM2 and its non-metabolic transcriptional regulatory role in the nucleus. Moreover, *Gf* extract suppressed LDHA expression and lactate production (56, 57), effectively disrupting the pro-inflammatory, lactate-enriched microenvironment that sustains M1 polarization at the metabolic terminus.

This multi-target, synergistic inhibition of the glycolytic pathway, from the “entry” (GLUT1) to the “exit” (LDHA) points, suggests that *Gf* extract dismantles the metabolic foundation of M1 macrophages. Its mode of action mirrors that of the well-characterized glycolytic inhibitor 2-DG, but with potential advantages in safety and efficacy due to its natural, multi-component composition (58).

Importantly, metabolic flux analysis provided additional functional insights into the immunomodulatory effects of *Gf* extract. The core mechanism lies in the comprehensive suppression of absolute metabolic flux rather than merely altering the relative contributions of glycolysis and oxidative phosphorylation. In highly glycolysis-dependent THP-1 cells, *Gf* treatment significantly reduced absolute metabolic indicators, such as lactate production, without altering the proportional energy contribution of glycolysis. Similarly, in RAW264.7 cells, *Gf* extract effectively suppressed the M1 inflammatory phenotype but did not completely reverse the high glycolytic contribution mode. Instead, it imposed a “comprehensive metabolic brake” on overactivated macrophages by concurrently inhibiting both glycolysis and mitochondrial respiration. This mechanism—”suppressing flux without altering proportion”—represents a novel strategy for immunomodulation, wherein metabolic intensity is downregulated without fundamentally restructuring the metabolic architecture.

Despite these promising findings, certain limitations remain. As a chemically complex mixture, the specific active constituents of *Gf* extract responsible for its anti-ALD effects are yet to be identified. Furthermore, while the study demonstrated the coordinated inhibition of glycolytic enzymes, it remains unclear whether this is mediated through direct enzyme interactions or indirect regulation of upstream signaling pathways, such as Akt/mTOR/HIF-1α.

Future research should focus on: (1) bioactivity-guided fractionation to isolate and identify the core active components of *Gf* extract; (2) elucidating upstream signaling mechanisms governing glycolytic gene expression; and (3) evaluating stage-specific efficacy across ALD progression to determine optimal therapeutic windows.

## 5 Conclusion

Collectively, this work establishes *Gf* extract as an effective agent against alcoholic liver disease, acting through coordinated suppression of key glycolytic molecules to reverse M1 macrophage metabolic reprogramming and attenuate pro-inflammatory activation. The conserved effect across species underscores its mechanistic relevance and translational potential, providing a solid foundation for developing macrophage glycolysis-targeted strategies for ALD.

## Data Availability

The data presented in the study are deposited in the NCBI Sequence Read Archive (SRA) repository: https://www.ncbi.nlm.nih.gov/sra/?term=PRJNA1418400.

## Supplementary material

The Supplementary Material for this article can be found onlineat: https://www.frontiersin.org/articles/10.3389/fimmu.2026.1772592/full#supplementary-material

## Glossary

*Gf*: *Gomphus floccosus* (Schw.) Sing.
ALD: Alcoholic liver disease
UHPLC-Q-Exactive Orbitrap HRMS: Ultra-High-Performance Liquid Chromatography coupled with Q-Exactive Orbitrap High-Resolution Mass Spectrometry
ALT: alanine aminotransferase
AST: aspartate aminotransferase
TC: total cholesterol
TG: triglycerides
H&E: hematoxylin-eosin staining
IHC: Immunohistochemistry
mIHC: multiplex immunohistochemistry
DEGs: differentially expressed genes
GO: gene ontology
KEGG: kyoto encyclopedia of genes and genomes
BP: biological process
CC: cellular component
MF: molecular function
ELISA: enzyme linked immunosorbent assay
IL-1*β*: interleukin-1*β*
IL-6: interleukin- 6
IL-10: interleukin-10
WB: Western blot
RT-qPCR: Real-time quantitative polymerase chain reaction
FBS: Fetal Bovine Serum
LPS: lipopolysaccharide
PMA: phorbol 12-myristate 13-acetate
CCK8: Cell Counting Kit-8
ICC: Immunocytochemistry
