# Supplementary figures and images for: Gomphus floccosus (Schw.) Sing. extract attenuates alcoholic liver disease by suppressing macrophage glycolysis and M1 polarization
Source: Front Immunol. 2026 Feb 17;17:1772592. doi: 10.3389/fimmu.2026.1772592 (PMC12953122; doi:10.3389/fimmu.2026.1772592)

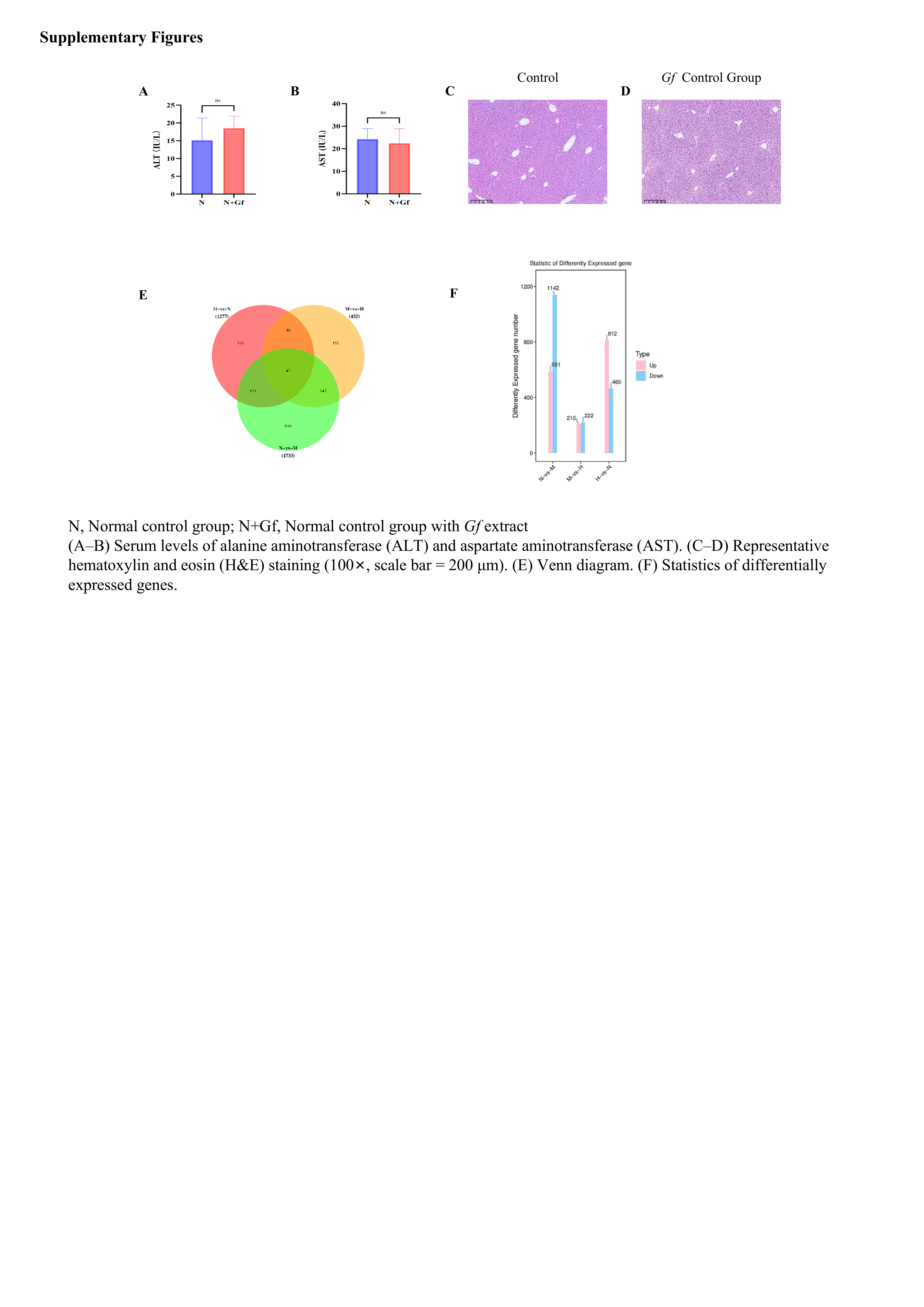

Supplement: Supplementary file 1 [file Image1.tif]
